# Visualization of elemental distributions and local analysis of element-specific chemical states of an *Arachnoidiscus* sp. frustule using soft X-ray spectromicroscopy

**DOI:** 10.1371/journal.pone.0243874

**Published:** 2020-12-16

**Authors:** Tomoko Ishihara, Takuo Ohkochi, Akinobu Yamaguchi, Yoshinori Kotani, Masaki Oura

**Affiliations:** 1 Soft X-ray Spectroscopy Instrumentation Team, Physical and Chemical Research Infrastructure Group, Advanced Photon Technology Division, RIKEN SPring-8 Center, Sayo-gun, Hyogo, Japan; 2 Spectroscopic Analysis Group II, Spectroscopy and Imaging Division, Japan Synchrotron Radiation Research Institute (JASRI), Sayo-gun, Hyogo, Japan; 3 Laboratory of Advanced Science and Technology for Industry (LASTI), University of Hyogo, Ako-gun, Hyogo, Japan; Lawrence Berkeley National Lab, UNITED STATES

## Abstract

Using soft X-ray (SX) spectromicroscopy, we show maps of the spatial distribution of constituent elements and local analysis of the density of states (DOS) related to the element-specific chemical states of diatom frustules, which are composed of naturally grown nanostructured hydrogenated amorphous silica. We applied X-ray photoemission electron microscopy (X-PEEM) as well as microprobe X-ray fluorescence (μXRF) analysis to characterize the surfaces of diatom frustules by means of X-ray absorption spectroscopy (XAS) and X-ray emission spectroscopy (XES). We successfully demonstrated that SX spectromicroscopy is able to participate in potential observation tools as a new method to spectroscopically investigate diatom frustules.

## Introduction

Diatoms are a major group of eukaryotic unicellular microalgae with characteristic cell walls composed of a composite of organic material and nanoporous hydrogenated amorphous silica, referred to as frustules [1–3]. Similar to terrestrial plants, diatoms play a crucial role in carbon fixation and generate approximately 20% of the oxygen produced on Earth each year by photosynthesis [4, 5]. In addition to existing in oceans, diatoms are widely found in lakes, marshes, rivers and some hot springs worldwide, wherever there is sufficient sunlight and nutrients [6]. In addition to being photosynthetic organisms, diatoms are also thought to play a key role in the production of useful substances, and research on various applications (see, for example, a review by Mishra *et al*. [7]) is being widely conducted by taking advantage of the potential of diatoms [8–11].

Diatom frustules are created with high precision, with a size of tens of nanometers, and show an extraordinary variety of three-dimensional shapes and structures [12]. Diatoms can be roughly classified into two distinct shapes: most of them are broadly bilaterally symmetric (pennate diatoms), while a few of them are radially symmetric (centric diatoms). Such diverse diatoms, with almost 10^5^ different species, range in size from a few micrometers to a few hundred micrometers. Thus, unique frustule morphologies are very important for the taxonomic study of diatoms, and a microscopic observation tool is indispensable for diatom taxonomy. Therefore, progress in our understanding of such diatoms is closely related to the development of microscopes.

Since the first observation of diatoms by using a simple optical microscope more than 250 years ago, optical microscopy has long been the dominant method for observing diatoms. However, since the invention of the electron microscope in the first half of the 20th century, many morphological studies have been conducted on diatoms, especially on the micro-/nanostructure of their frustules. Currently, to examine the micro-/nanostructural features of diatom frustules, various microscopic and spectromicroscopic methods, including optical microscopy, transmission electron microscopy (TEM), scanning electron microscopy (SEM) [3, 12–21], atomic force microscopy (AFM) [12, 14–16, 19], transmission X-ray microscopy (TXM) [22, 23], microprobe X-ray fluorescence (μXRF) analysis [24, 25], and infrared (IR) microspectroscopy [3], are used to investigate diatoms.

In electron microscopy, *i*.*e*., TEM and SEM, element-specific information can be obtained by incorporating energy dispersive spectrometry (EDS) and electron energy loss spectrometry (EELS). For instance, SEM-EDS/TEM-EDS allows us to map the elemental distribution in a diatom frustule and to gain insight into their chemical compositions by analyzing the relevant maps. TEM-EELS, on the other hand, allows local element-specific analysis [20] and the ability to acquire spectral information on the density of states (DOS) near the lowest unoccupied molecular orbital (LUMO) of the element of interest. AFM has allowed us to obtain a detailed understanding of the micro-/nanostructural features of frustules [12, 14, 15, 19]. Loic *et al*. successfully elucidated that the silica wall typically consists of three overlapping porous layers, *i*.*e*., *areola*, *cribrum* and *cribellum* [12, 14, 15]. In the last decade, synchrotron radiation (SR)-based μXRF analysis has been successfully applied to visualize the elemental distribution in diatom frustules [24, 25]. In particular, de Jonge *et al*. demonstrated that tomographic μXRF analysis has high potential to quantify the three-dimensional distributions of the elements in freshwater diatoms at 400 nm resolution [24]. Similarly, Hitchcock *et al*. applied μXRF analysis combined with scanning TXM to study environmental materials by measuring the X-ray absorption spectroscopy (XAS) spectrum of *Acidovorax* sp. strain BoFeN1 [26].

Very recently, we developed two SR-based soft X-ray (SX) spectromicroscopy techniques [27–29] at the SX undulator beamline BL17SU of SPring-8. One is X-ray photoemission electron microscopy (X-PEEM) [27], with a maximum spatial resolution below 100 nm (36.7 nm) with the use of an SX beam (UV lamp) as an excitation source. The other is scanning SX spectromicroscope [28, 29], which utilizes the focused SX-beam as an excitation source and two-dimensionally scans the sample to carry out the μXRF analysis with a spatial resolution on the submicron scale. In the present study, we applied both of these SX spectromicroscopies to investigate diatom frustules for the first time.

In this paper, we report typical results of experiments using two SX spectromicroscopies, *i*.*e*., X-PEEM and μXRF analysis, to visualize the elemental distributions and to carry out local spectroscopic analysis for the DOS related to the element-specific chemical states of centric diatom (*Arachnoidiscus* sp.) frustules by means of XAS and X-ray emission spectroscopy (XES).

## Materials

**Fig 1:**
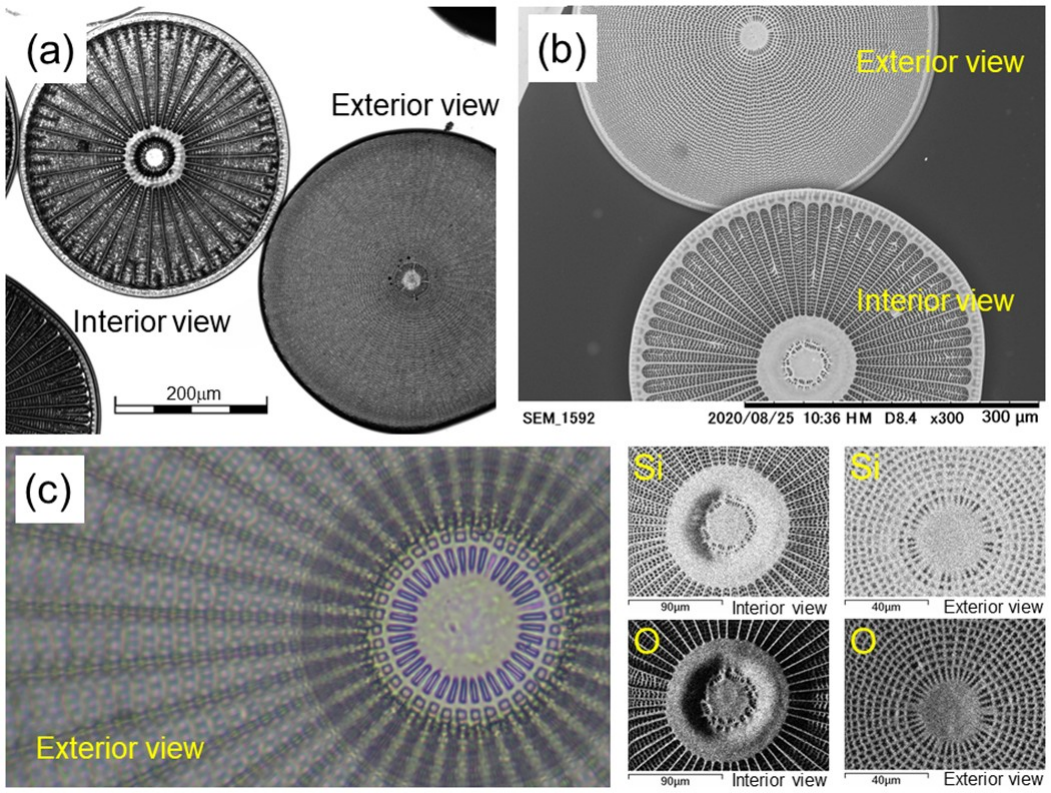
Micrographs of the frustule sample of *Arachnoidiscus* sp. (a) Interior and exterior views of frustules obtained by using a laser scanning digital microscope. (b) Exterior and interior views of frustules visualized by SEM. (c) Exterior view of a frustule near the center part obtained by an optical microscope. Four elemental distribution maps visualized by SEM-EDS are also shown for Si and O in the lower right corner.

Several single valves of *Arachnoidiscus ornatus* [13] frustules, which were washed and dehydrated with alcohol, were purchased [30] for use in the present study. The sizes of the frustules ranged between 230 and 400 μm in diameter. The frustules were dried just before primary observation using an optical microscope, a laser scanning digital microscope (Olympus LEXT OLS4100) and SEM-EDS (Hitachi TM3030Plus tabletop microscope). In Fig 1, we show some typical micrographs of the *Arachnoidiscus* sp. frustules taken by using (a) a laser scanning digital microscope (see also S1 and S2 Figs), (b) SEM (See also S3 Fig) and (c) an optical microscope. In the lower right corner of Fig 1, four elemental distribution maps recorded near the center of the frustule (both of the interior and exterior sides) are shown for silicon (Si) and oxygen (O).

## Methods

SX spectromicroscopic experiments on the diatom frustule samples were performed at the carry-in station, where two end-stations utilized for the present study could be easily switched by using the slide rails of the b-branch of beamline BL17SU [31–34] at SPring-8. Details of the end-stations, *i*.*e*., the surface-sensitive X-PEEM and bulk-sensitive scanning SX spectromicroscope, were described elsewhere [27–29]. The surface and bulk sensitivities of these techniques originate from their principles of measurement. X-PEEM, which is classified as a full-field microscopy using an electrostatic lens system, is based on the photon-IN/electron-OUT measurement scheme of measuring electrons to generate an image of the sample surface, whereas scanning SX spectromicroscope relies on the photon-IN/photon-OUT measurement scheme to generate an image of the sample surface. Thus, the probing depths are estimated to be a few nanometers and a few hundred nanometers for X-PEEM and scanning SX spectromicroscope, respectively.

The frustule samples were prepared for detailed SR-based observations using two spectromicroscopy techniques. Since X-PEEM detects photoelectrons or secondary electrons emitted from the outermost surface layer, *e*.*g*., a few nanometers, it is difficult to examine insulating materials, such as the frustule of a diatom, which is mainly composed of amorphous silica. Thus, the frustule samples were mounted on an indium sheet, and to avoid a surface charging effect, platinum (Pt) with a thickness of a few nanometers was sputtered to coat the surface. In the S4 Fig, we show typical examples of X-PEEM images affected by the charging effect. Then, the frustule samples were introduced into the target chamber of the X-PEEM apparatus and evacuated to ultrahigh vacuum conditions for observation. For the μXRF analysis using scanning SX spectromicroscope, on the other hand, the frustule samples were attached to double-sided conductive carbon tape stuck on a copper plate, and for the observation, the sample plate was mounted on high-precision actuators in the vacuum chamber of the apparatus for two-dimensional scanning. Scanning SX spectromicroscope also enables us to observe wet samples under a helium atmosphere (see, for example, S5 Fig), but in the present study, we carried out all the measurements under low-vacuum (~10 Pa) conditions. Furthermore, because the present apparatus works based on the photon-IN/photon-OUT measurement scheme, we were able to observe the frustule samples without a platinum coating.

In the SR-based observations, the incident photon energy was calibrated by using a hemispherical electron energy analyzer installed downstream of the present apparatuses. We measured the Au 4f_7/2,5/2_ photoelectron spectra as well as the near-valence spectra, including the Fermi edge, to precisely calibrate the photon energy. To obtain the 1860 eV focused SX-beam for the experiment using scanning SX spectromicroscope, we needed to operate the focusing optics, *i*.*e*., the Fresnel zone plate (FZP), of the spectromicroscope to achieve third-order diffraction, but we operated the system with first-order diffraction for the 530–600 eV focused SX-beam.

We should note here that radiation damage is always a problem in SX spectromicroscopy, especially for biological and polymer samples. To advance the microspectroscopic study of polymer samples, Wang and Hitchcock *et al*. quantitatively evaluated the radiation-induced damage of polymer samples by using scanning TXM and X-PEEM [35–37]. We also study the radiation-induced damage of resinous samples here at SPring-8 [38, 39]. Thus, we expect to determine how serious the radiation-induced damage is, especially for near-future experiments on living attached diatoms, using scanning SX spectromicroscope. In such cases, we will need to acquire all the data with a quick-scan, the so-called on-the-fly, measurement scheme before the sample is seriously damaged by radiation. In the present study, however, we do not pay much attention to the issue of radiation-induced damage since the diatom frustule sample used in the present study was composed of amorphous silica and dried.

## Results and discussion

### X-PEEM observation

**Fig 2:**
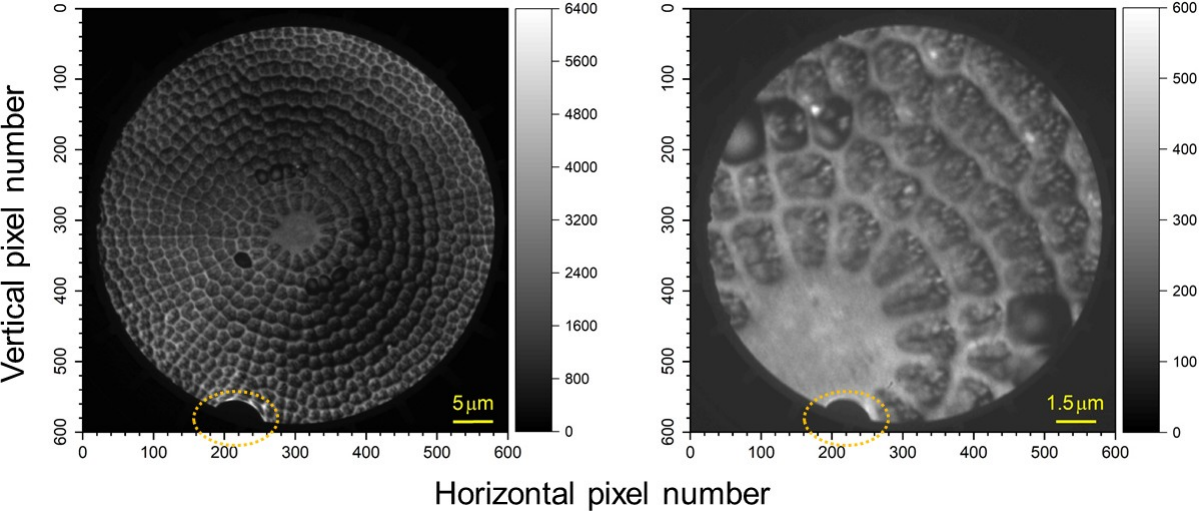
X-PEEM images of a diatom frustule (exterior view). X-PEEM images were obtained at two different FOVs (left: 50 μm, right: 15 μm). A UV-lamp was used as the excitation source. The contrast of the image is related to the local variations in the electron emission rate of the Pt-coated sample surface. Brighter parts correspond to regions having higher electron emission rates with lower work functions. The dotted orange ellipses near the bottom of the images indicate the image distortion caused by a problem in the image intensifier.

First, we carried out observations of diatom frustules with two different fields of view (FOVs) by using a UV lamp as an excitation source. Fig 2 shows the resultant X-PEEM images. Each image was generated by summing 100 consecutive X-PEEM images, where the dwell time for a single image was five seconds. One hundred X-PEEM images were collected for image blurring and then summed to obtain the final image. In both images, we can observe identical image distortion near the bottom of the images, indicated by the dotted orange ellipse, which was caused by a problem in the image intensifier. In these images, the contrast of the image is related to local variations in the electron emission rate of the Pt-coated sample surface. For the larger-FOV image, *i*.*e*., 50 μm (left panel), there seems to be an intensity variation at a coarse spatial scale. This might be due to uneven coating of Pt or the degree of curvature of the diatom frustule. In particular, the former will give rise to a position-dependent difference in elimination of the charging effect, which results in an intensity variation. For the smaller-FOV image, *i*.*e*., 15 μm (right panel), the X-PEEM apparatus was operated with a spatial resolution on the order of sub-100 nm. Clearly, there are some bright spots in the image that are considered to originate from the sample itself or from the dust attached on the frustule surface, not from instrumental factors such as bad pixels.

**Fig 3:**
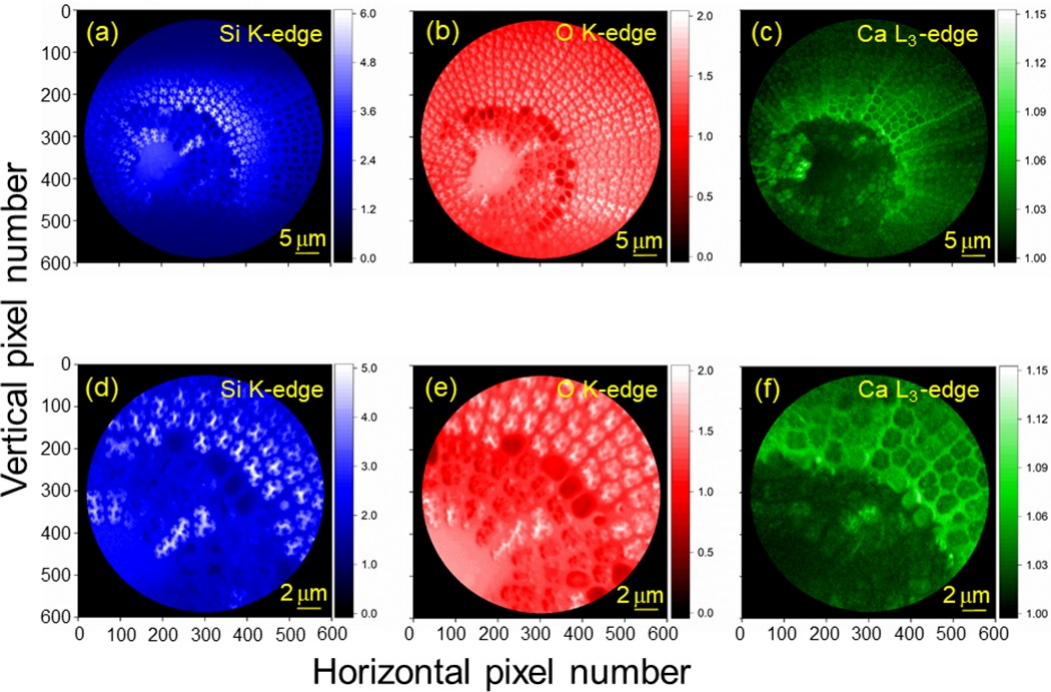
Contrast-Enhanced (CE) X-PEEM images of a diatom frustule (exterior view). The CE X-PEEM images were reproduced for (a & d, blue) Si-K, (b & e, red) O-K and (c & f, green) Ca-L_3_ edges. These CE X-PEEM images were recorded using SR as an excitation source and reproduced from the images acquired above the absorption edges, or the top of the resonance, divided by those recorded below the edges. The brightness, *i*.*e*., the contrast, of each image is related to the optical density of the target material.

Second, we performed an SR-based X-PEEM observation for the same frustule samples. We tuned the SX-beam energy to be just above the K absorption edges of relevant elements to selectively excite the Si and O contained in the sample. These elements are the main constituents of diatom frustules composed of silica. Additionally, the SX-beam energy was tuned to be above the L_3_ absorption edge to preferably excite calcium (Ca), which is considered a trace element originating from biological residues or present in the frustule as a bioincorporated mineral [18, 21, 25]. Two images were then recorded for each element at the excitation energies above or at the top of the resonance and below the absorption edge to generate a contrast-enhanced (CE) X-PEEM image. The CE images were reproduced from the images acquired above the absorption edges, or the top of the resonant excitation, divided by those recorded below the edges. The resultant CE X-PEEM images are shown in Fig 3, and they are free from inhomogeneity due to the work function related to the surface morphology or local variations in the electron emission rate of the Pt-coated sample surface since the division of two images cancels out the position dependence. Thus, the brightness of the CE X-PEEM images is related to the optical density of the target material [40] for the corresponding excitation energies. Some regions were observed to be slightly affected by the charging effect, as we show for some examples in the S6–S8 Figs. In S6–S8 Figs, the X-PEEM images recorded at the excitation energies above or at the top of the resonance (peak) and below (pre) are shown together with the CE X-PEEM images (div), where div equals peak/pre. As shown in Fig 3, brighter regions observed in the Si and O images are very similar to each other because these elements are both main constituents of silica diatom frustules. Especially in Fig 3b & 3e, we can see that the primary *costae* radiating from the central part are dim red. On the other hand, slightly brighter regions in the Ca image are seen to be opposite to the cases of Si and O. We can easily recognize the *costae* in bright green in Fig 3c & 3f. As seen in Fig 3c & 3f, Ca is observed to show the contrast reflecting the surface morphology of the frustule, although the statistics for Ca L_2,3_-XAS (shown below in Fig 5) are quite poor. Thus, it seems to be quite natural that Ca can be bioincorporated into silica during the formation of frustules.

**Fig 4:**
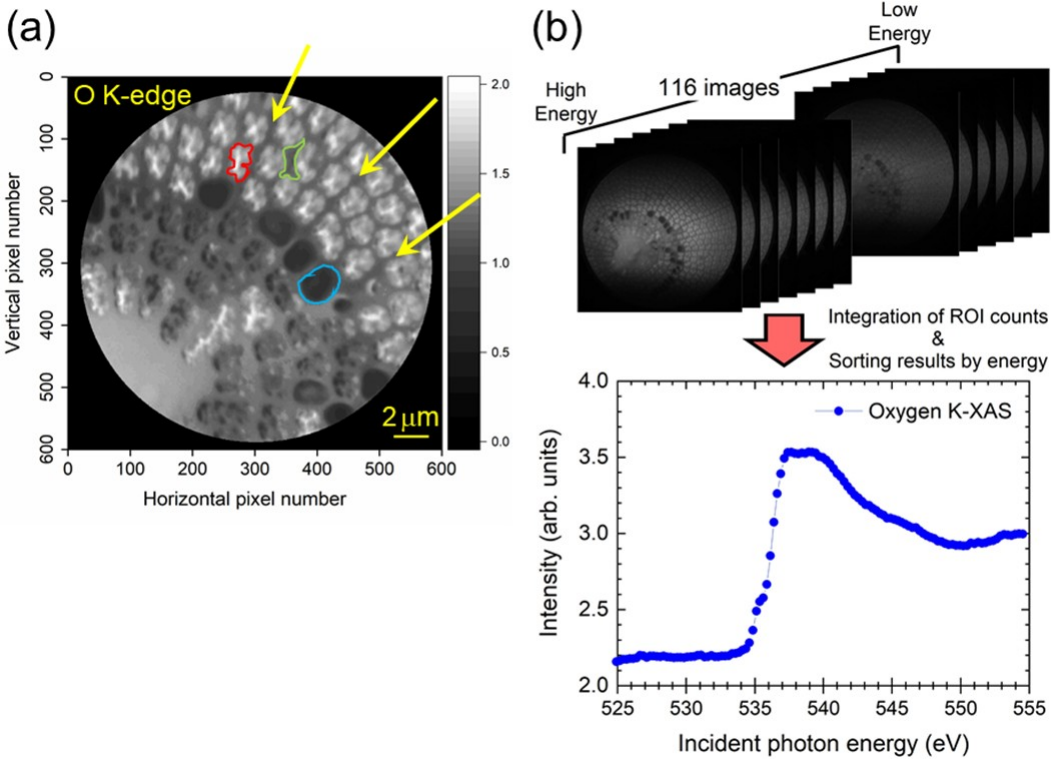
CE X-PEEM image and the scheme for recording the K-XAS spectrum. (a) CE X-PEEM image, shown in grayscale, of a diatom frustule recorded at the O K absorption edge. Three characteristic ROIs, marked by the areas enclosed with red, green and blue lines, were selected to extract the local XAS spectra shown below in Fig 5. The yellow arrows indicate the primary *costae*. The red ROI corresponds to the pores (*areola*) [3, 13, 19]. (b) Scheme for recording the O K-XAS spectrum using a series of X-PEEM images acquired by sweeping the energy of the incident photon beam across the O K-edge (upper half). The total number of images was 116. The O K-XAS spectrum (lower half) was constructed by integrating the ROI intensity, where the ROI was chosen to be the whole FOV in this case, normalizing the integrated intensity by the area of the ROI and sorting the results by incident photon energy.

To acquire the K-XAS spectrum to study the local electronic structure related to the target O element, we carried out a series of X-PEEM observations by sweeping the excitation energy across the O K absorption edge. Each image was taken with a 20 μm FOV, and the dwell time per single image was five seconds. We recorded 116 X-PEEM images in total. To generate the K-XAS spectrum, we set the ROIs, where ROI stands for the region of interest, at arbitrary locations inside the X-PEEM image, as shown in Fig 4a, and normalized the ROI intensity recorded in each image using the area of the ROI. Then, we sorted the normalized intensities by energy to generate the K-XAS spectrum, as shown in Fig 4b. The resultant O K-XAS spectrum shown in Fig 4b is definitely very similar to that of SiO_2_ (glass) reported in a previous paper [41, 42].

**Fig 5:**
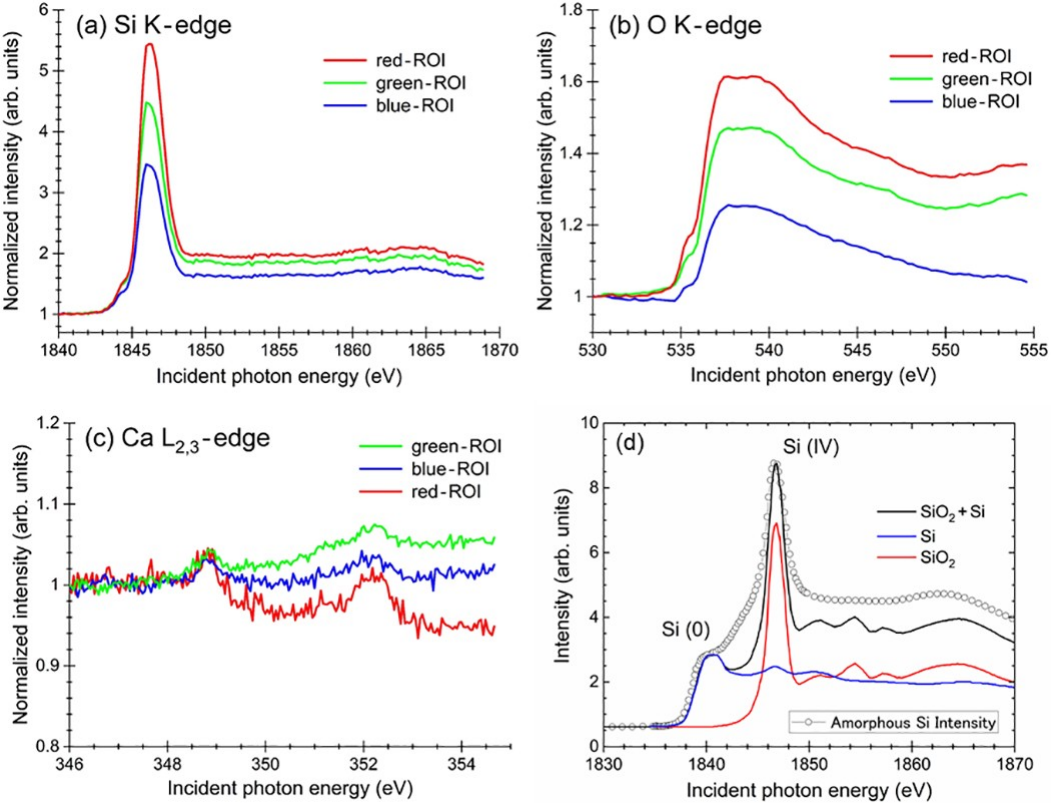
XAS spectra constructed from the X-PEEM images. XAS spectra recorded across the absorption edges of (a) Si K, (b) O K and (c) Ca L_2,3_. (d) Reference Si K-XAS spectra of Si and SiO_2_ as well as amorphous SiO. The curves shown in Fig 5d are reproduced from Fig 1 presented in the internal report R1405 of the Synchrotron Radiation Center of Ritsumeikan University (written in Japanese) [43].

By employing such an XAS spectrum measurement scheme, we carried out local XAS measurements on each element by employing three different ROIs to examine the position-dependent electronic structure in the frustule sample. As shown in Fig 4a, we selected three ROIs, red, green and blue ROIs, as shown in the figure. For the cases of Si and O, the red ROIs correspond to the bright blue and bright red regions, respectively, as indicated in Fig 3a, 3d, 3b & 3e. Similarly, the green ROI is related to the medium-blue and medium-red regions. The blue ROI represents the dark blue and dark red regions in Fig 3, possibly the portion affected by the charging effect (see also S5–S7 Figs). For the case of Ca, on the other hand, the situation is slightly different from the cases of Si and O described above. The green ROI corresponds to the bright-green region shown in Fig 3c & 3f, the blue ROI represents the region in medium-green, and the red ROI is related to the region in medium-green. In the case of Ca, the blue ROI seems to be less affected by the charging effect because of the lower signal intensity compared to the cases of Si and O. By using these ROIs, we reconstructed the XAS spectra, as shown in Fig 5, for each target element.

The XAS spectra in Fig 5a–5c show that the elements contained in each ROI seem to be in a similar chemical state, where the spectral shapes of the red, green, and blue curves in each panel are quite similar. Only the intensities are different, as shown in the figure, due to the differences in the corresponding optical density for each portion of the target material. In Fig 5d, we also show the typical Si K-XAS spectra of Si and SiO_2_ as well as amorphous Si for reference [43]. The Si K-XAS spectra shown in Fig 5a indicate the typical spectral shape of amorphous silica (a-SiO_2_), as previously reported [44]. According to their assignments [44], a pronounced peak at 1847 eV is attributable to the transition of Si 1s electrons to the antibonding t_1u_ orbital (Si 3p-like state), and a weak peak observed at 1844 eV is attributed to the dipole-forbidden transition of Si 1s → 3s. As shown in Fig 5d, we can also expect that amorphous Si monoxide contributes to the weak peak at 1844 eV, and furthermore, a broad bumpy peak at approximately 1865 eV is recognizable, as reported in a previous paper [43, 45].

As we mentioned above, the O K-XAS spectra shown in Fig 5b were quite similar to those of SiO_2_ (glass) reported in a previous paper [41, 42]. As in the case of the Si K-XAS spectra, the chemical states were found to be similar in each ROI. As shown in Fig 5c, the Ca XAS spectra at the L_2,3_ edges were definitely observed, although their intensities were very weak. Each spectrum consists of two peaks attributable to the dipole-allowed transitions of Ca 2p electrons, *i*.*e*., 2p_1/2_ and 2p_3/2_, into 3d-like final states [46]. The absolute energies of these two peaks observed in the Ca L_2,3_-XAS spectra were measured to be slightly higher, *e*.*g*., approximately 0.2 eV, and the energy difference between the two peaks, *e*.*g*., approximately 3.4 eV, was similar to that for CaO, previously reported by Himpsel *et al*. [46]. As we mentioned previously, Ca can be considered to be bioincorporated into silica as Ca oxide during the formation of frustules. Therefore, it might be considered that Ca atoms are contained in the surface of the present frustule by forming CaO.

### μXRF analysis

**Fig 6:**
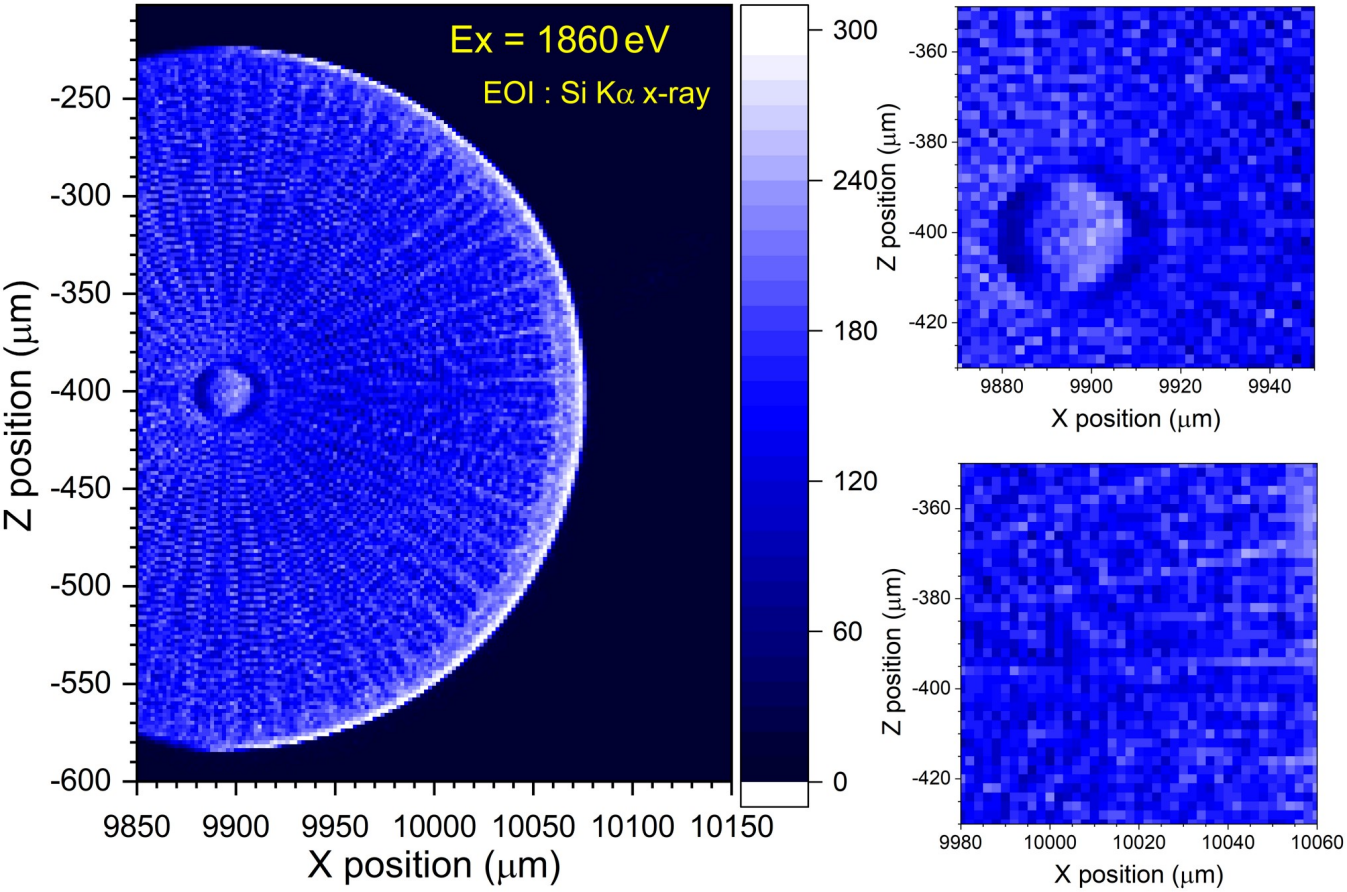
A μXRF single-element map of Si visualized by measuring Si Kα X-rays from the exterior surface of an *Arachnoidiscus* sp. frustule. The μXRF single-element map was recorded using the 1860 eV focused SX beam. Due to the geometry of the measurement, the left half of the frustule shows less contrast. This μXRF map was recorded with a 2 μm pixel size and an exposure time of one second for one pixel, where EOI stands for energy of interest.

As a first step for the SR-based μXRF analysis of diatom frustules using a scanning SX spectromicroscope, we carried out μXRF single-element mapping to roughly visualize the surface morphology of the frustule by measuring the Si Kα X-rays excited by the 1860 eV focused SX beam using a silicon drift detector (SDD). Fig 6 shows the resultant μXRF single-element map of Si obtained from the frustule sample of *Arachnoidiscus* sp. measured with a 2 μm pixel size and an exposure time of one second for one pixel. Similar to the case of the X-PEEM image, the brightness of the single-element map is related to the optical density of the target material of silica, that is, the concentration of Si in the frustule surface. It should be noted, however, that the left part of the map seems to be slightly darker than the right part of the map. This is due to the arrangement of the detection system for the present scanning SX spectromicroscope [28]. In this arrangement, a rugged structure on the frustule surface causes a shadowing effect during sample scanning because of a grazing exit angle, *i*.*e*., a small take-off angle for fluorescence X-ray detection.

**Fig 7:**
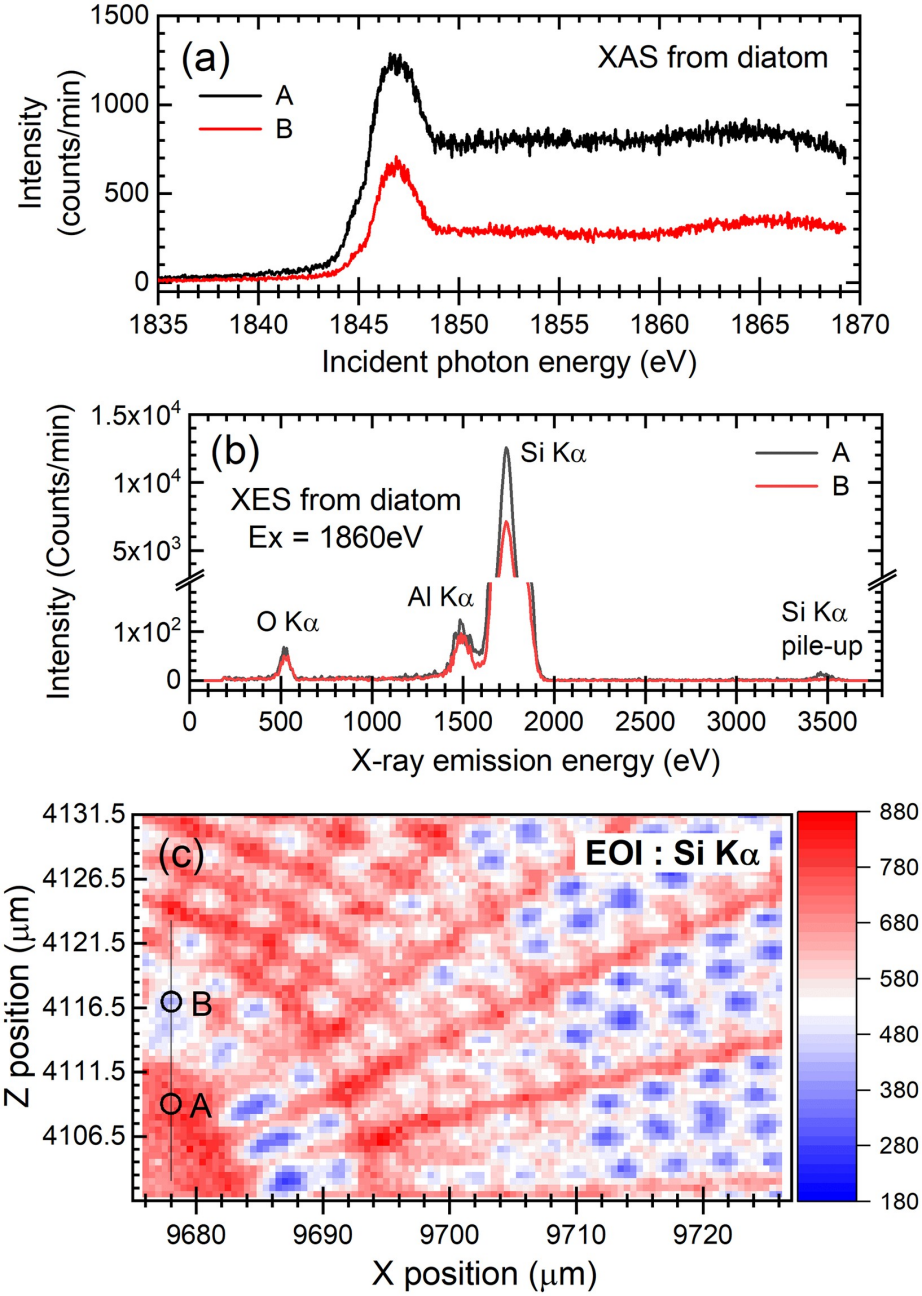
Local XAS/XES spectra and the μXRF single-element map of a diatom frustule. (a) Si K-XAS spectra and (b) fluorescence X-ray spectra of a frustule of *Arachnoidiscus* sp. recorded at two different positions, *i*.*e*., A and B, marked in the bottom panel of (c). (c) μXRF single-element map of Si obtained from near the center of the exterior surface of the frustule of *Arachnoidiscus* sp. using the 1860 eV focused SX-beam. The μXRF single-element map was recorded using the PFY method with a 0.5 μm pixel size and an exposure time of 1 second for one pixel, where EOI stands for energy of interest.

Second, in Fig 7, we show the results of Si K-XAS spectra (a) and fluorescence X-ray spectra (b) obtained from the local areas A and B marked in the μXRF single-element map (c) of an *Arachnoidiscus* sp. frustule excited by using the 1860 eV focused SX-beam. Si K-XAS spectra were measured by the partial fluorescence yield (PFY) method, where the number of Si Kα X-rays was counted as a function of the excitation energy. Each Si K-XAS spectral shape shown in Fig 7a was observed to be essentially the same as those obtained by the X-PEEM (Fig 5). The spectrum consists of a pronounced peak at 1847 eV, a weak peak at approximately 1845 eV and a broad bump at approximately 1865 eV, as shown in Fig 5. Thus, the resultant Si K-XAS spectra measured using the μXRF-PFY method also confirmed that the frustule is composed of amorphous silica (a-SiO_2_). The intensity of each curve, *i*.*e*., A and B, correspond to the amplitude of the μXRF single-element map shown in Fig 7c. We also noticed that the pronounced peak at 1847 eV is considerably broader than that in Fig 5a. The full width at half maximum (FWHM) of the former was observed to be approximately 2.5 eV, and that of the latter was approximately 1.9 eV. The additional broadening was considered to originate from the method used to acquire the XAS spectrum. In the case of XAS by X-PEEM, the spectrum was measured by integrating the ROI intensity of the X-PEEM image, followed by normalization using the area of the ROI as a function of the excitation energy. We collected the secondary electrons emitted from the sample surface with a fairly high collection efficiency. Thus, it is very similar to the total electron yield (TEY) method, and the resultant FWHM of the XAS peak is mainly determined by the bandpass of the beamline monochromator and the natural width of the core-hole state. On the other hand, in the case of XAS by μXRF analysis with the PFY method, we count the characteristic X-rays emitted near the sample surface by using the fairly large energy resolution SDD, *e*.*g*., approximately 80 eV at the O Kα X-ray energy. As has been demonstrated by Hämäläinen *et al*. [47], the resolution of the detector used for the PFY method causes additional broadening of the resultant XAS spectrum. Therefore, the additional broadening observed in the XAS spectrum in Fig 7a is considered to originate from the fairly large energy resolution of the SDD used in this study.

The fluorescence X-ray spectra, measured by means of X-ray emission spectroscopy (XES), shown in Fig 7b, were recorded by using the 1860 eV focused SX-beam. The measurement time for each fluorescence X-ray spectrum was five minutes. The relative intensity between Si and O was observed to be quite different, although the stoichiometry of SiO_2_ is 1:2. This difference can be explained by the differences in the photoabsorption cross sections of Si and O at an excitation energy of 1860 eV [48] and the differences in the fluorescence yield of each element, *e*.*g*., 0.05 for Si and 0.0083 for O [49], and the detection efficiency of the present SDD for Si (~ 68%) and O (~ 30%) Kα X-rays. All the XES spectra show a weak peak of Al Kα X-rays on the low-energy side of the pronounced peak of the Si Kα X-rays. The appearance of the Al Kα X-ray peak is probably due to spectral contamination by the Al coating on the Si_3_N_4_ membrane window of the SDD. The Al coating can be excited by the elastically scattered 1860 eV SX-beam as well as the pronounced Si Kα X-rays emitted from the frustule sample. Thus, we conclude that the weak Al Kα X-ray peak originates from the coating of the SDD window, not the impurity contained in the frustule.

For the μXRF single-element map of Si shown in Fig 7c, we should note here that the intensity map seems to be observed inversely to the case of the CE X-PEEM image. As shown in the figure, the regions of the primary *costae* show high intensity, and the regions of the pores (*areola*) indicate low intensity. This situation is opposite to the case of the CE X-PEEM, as shown in Fig 3a & 3d. Possible explanations can be considered as follows:

1. *The sensitivity of each spectromicroscope is essentially different*, *as we have mentioned in the Methods*.
  The contrast in the surface-sensitive X-PEEM image is related to the local variations in the electron emission rate of the sample surface as well as the optical density of the target material contained in the surface region. Elements of interest contained in a few nanometers from the frustule surface can be measured by the X-PEEM. A thick insulating material, such as the primary *costae*, may sometimes affect the image due to the charging effects, resulting in a deterioration of the counting efficiency. On the other hand, bulk-sensitive μXRF contrast is relevant to the optical density of the target material in the frustule sample and is not affected by the charging effects. The probing depth in μXRF analysis reaches the submiron scale. Thus, the dense portion, *i*.*e*., the primary *costae*, gives rise to high signal intensity.
2. *The sample geometry of each spectromicroscope is different*.
  For the X-PEEM, the SX-beam irradiates the sample surface with an incident angle of 30 degrees, and the emitted secondary electrons are measured at an almost normal emission geometry; thus, the inside of the pores (*areloa*) can be easily observed when the SX-beam effectively reaches the pores. For the case of μXRF, on the other hand, the focused SX-beam irradiates the sample surface with an incident angle of approximately 70 degrees, and the emitted fluorescent X-rays are detected in the geometry of a grazing exit angle of 20 degrees; thus, the fluorescent X-rays emitted from the portions inside the pores are hardly observed due to shadowing effects. Thus, we understand that the regions of primary *costae* are clearly observed with high counting efficiency, and the regions of the pores (*areola*), especially inside the pores, are measured with lower counting efficiency for the μXRF analysis than for the X-PEEM.

**Fig 8:**
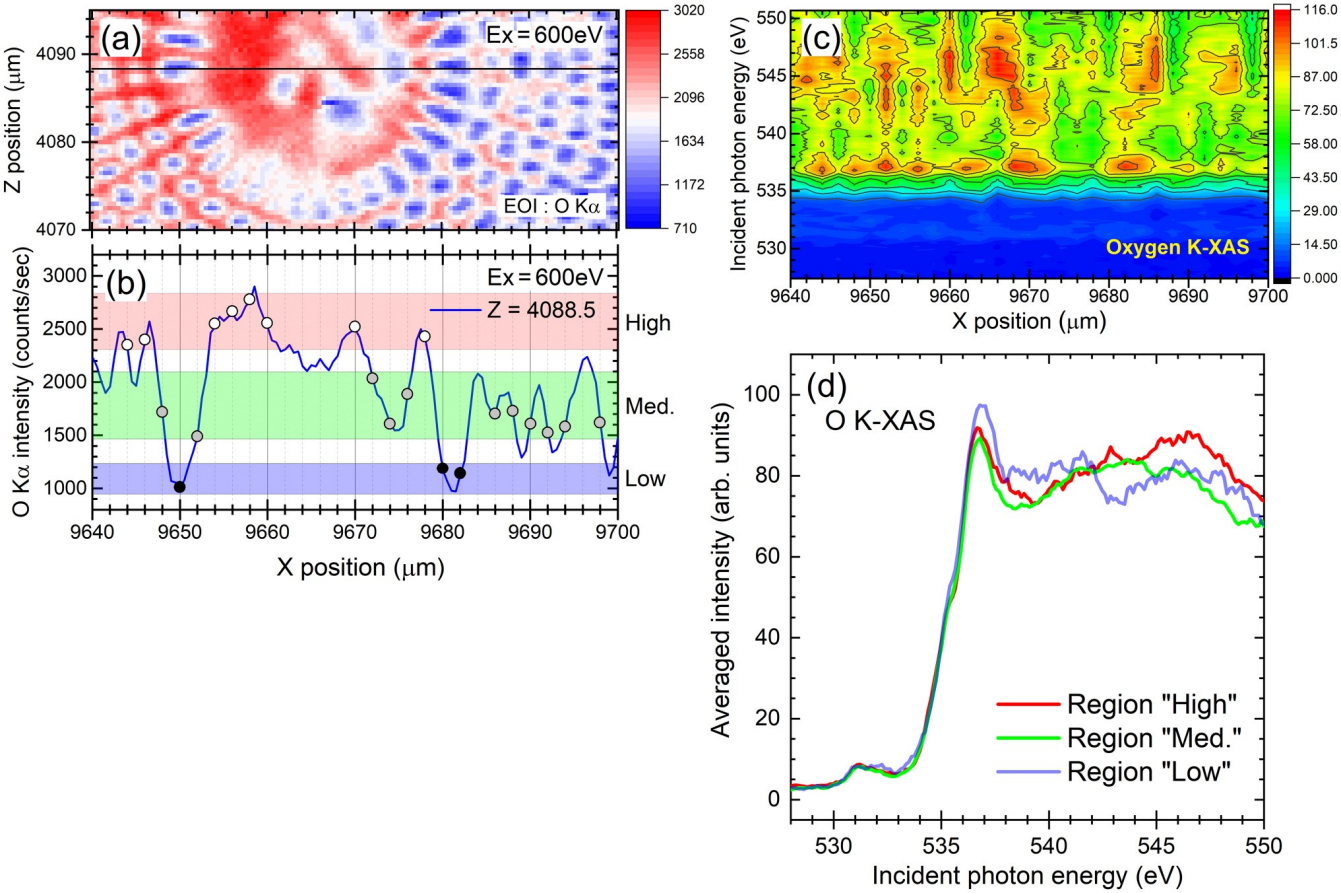
The μXRF single element map of O and position-dependent O K-XAS spectra. (a) The μXRF single element map of O visualized by measuring O Kα X-rays emitted from a frustule sample of *Arachnoidiscus* sp. using a 600 eV focused SX-beam. (b) The position-dependent intensity curve showing the number of O Kα X-rays measured around the near-center part, *i*.*e*., along the horizontal black line shown in panel (a). The curve is classified as “High”, “Med.” and “Low” according to the O Kα intensity recorded using the PFY method at the different positions. (c) Typical demonstration of visualizing the element-specific chemical-state distribution of oxygen obtained by measuring the O K-XAS spectra of the frustule sample of *Arachnoidiscus* sp. The O K-XAS spectra were recorded using the PFY method along the horizontal black line shown in panel (a). (d) The averaged O K-XAS spectra recorded at the regions classified as “High”, “Med.” and “Low”.

In Fig 8a, we show the μXRF single element map of O visualized by measuring O Kα X-rays emitted from the frustule sample of *Arachnoidiscus* sp. using the 600 eV focused SX-beam. The μXRF map was recorded with a 0.5 μm pixel size and an exposure time of 1 second for one pixel (see also S9 Fig). As shown in Fig 8b, we classified the position-dependent intensity of the O Kα X-rays measured using the PFY method into “High”, “Med.” and “Low” according to the O Kα intensity recorded at the different X positions along the horizontal black line shown in Fig 8a. We can easily recognize that the amplitude of the intensity curve clearly corresponds to the color map shown in panel (a). In Fig 8c, we demonstrate typical examples for visualizing the element-specific chemical-state distribution of oxygen contained in the frustule sample by measuring the position-dependent O K-XAS spectra at the near-center part, *i*.*e*., along the horizontal black line shown in panel (a), of *Arachnoidiscus* sp. Fig 8c and S10 Fig represent the position-dependent O K-XAS spectra shown in 2- and 3-dimensional views, respectively. As seen in the demonstration shown in Fig 8c and S10 Fig, the element-specific chemical state information for the local area can be visualized easily. We obtained the averaged O K-XAS spectra recorded at the classified regions, as represented in Fig 8d. These XAS spectra are averaged and not normalized. As in the case of the O K-XAS recorded by the X-PEEM, a steep rise at 535–536 eV due to the O K-edge jump is observed in each spectrum. Furthermore, we recognize the weak structure, *i*.*e*., the doublet peak, is observed at 531 and 532 eV in each curve. These small peaks are thought to be associated with nonbridging oxygen ions at 531 eV and with hydroxyl groups, *e*.*g*., Si-OH, whereas the main structure at the steep rise is associated with bridging oxygen, *e*.*g*., Si-O-Si [42].

Finally, as a prospect for the near future, we are developing a microflowing cell system to apply scanning SX spectromicroscope to the observation of living attached diatoms, such as *Arachnoidiscus* sp. If we can make the diatom attached to the Si_3_N_4_ membrane window of the microflowing cell system, we will be able to observe a state of attachment, especially an interface between the diatom and the membrane, through the Si_3_N_4_ window. Currently, we are testing a preliminary observation of the dried diatom frustule through the membrane window. In the near future, we would like to perform a trial measurement with the living attached diatom. As we have described in the Methods section, we should be aware of radiation damage when we observe living attached diatoms. We should record the map and the XAS spectrum by means of the quick-scan measurement scheme before the diatom is severely damaged by the focused SX-beam.

## Summary

We have demonstrated the capabilities of the present SX spectromicroscopy, *i*.*e*., X-PEEM and μXRF analysis by using a scanning SX spectromicroscope to spectroscopically characterize the diatom frustule with a spatial resolution below the submicron scale. Visualization of elemental distributions as well as local analysis of the density of states related to element-specific chemical states of the frustule of centric diatom, *Anachnoidiscus* sp., have been successfully performed for the first time under low-vacuum conditions. We could confirm that the micrographs obtained by means of SX spectromicroscopy are essentially equivalent to those obtained by optical microscopy as well as SEM. In addition to the visualization of elemental distributions, however, we could carry out further chemical-state analysis for the elements of the diatom frustule constituent.

As typical examples, we have shown here several experimental results on diatom frustules to demonstrate the capabilities of the present SX spectromicroscopy for exploring future studies. One can recognize that there are a wide variety of applications for various materials by utilizing the present SX spectromicroscopy. For the scanning SX spectromicroscope, by utilizing the flexibility around the sample area, we are also able to control the atmospheric conditions for the sample from low vacuum to helium atmosphere (see, for example, S5 Fig) or to change the sample condition by applying an external field to investigate the dynamics. To reconfirm their usefulness, we summarize their abilities, as shown in Table 1.

**Table 1:** Available photon source and energy range, achieved spatial resolution, and operation mode available for the X-PEEM and the scanning SX spectromicroscope installed at BL17SU of SPring-8.

Apparatus	Excitation sources	Photon energy range (eV)	Principle of microscope	Spatial resolution (nm)	Available operation mode
**X-PEEM**	UV^#1^	4.9 ^#2^	full-field	36.7	✧ Photoelectron imaging
	SR	250–2000		~100	✧ Secondary electron imagSing
					✧ Imaging ESCA^#5^
					(ΔE_K_ ~ 0.4 eV, E_K_ up to 1600 eV)
					✧ Local XAS analysis
					(consecutive imaging as a function of SX energy)
					✧ Local XMLD imaging
					(switching of horizontal and vertical polarization)
					✧ Local XMCD imaging
					(switching of helicity)
					✧ Specific chemical-state imaging
					(combined with resonant excitation)
					✧ Time-resolved nanospectroscopy
					(pump-probe method combined with a femtosecond pulsed-laser system)
					✧ k-space imaging
**Scanning SX spectro-microscope**	SR	400–756 ^#3^,	scanning	300 ~ 500 ^#3^,	✧ Local XES analysis
					✧ Local XAS analysis
		1200–2000 ^#4^		800 ~ 1200 ^#4^	(PFY method)
					✧ Elemental mapping
					(μXRF analysis up to 8 elements simultaneously)
					✧ Specific chemical-state mapping
					μXRF analysis combined with resonant excitation)

^#1^ ultraviolet (UV), compact discharge excitation source in the UV region.

^#2^ Main at 253 nm.

^#3^ 1^st^-order diffraction.

^#4^ 3^rd^-order diffraction.

^#5^ Electron spectroscopy for chemical analysis (ESCA).

In addition to Table 1, we also enumerate the features of each device installed at BL17SU, such as its advantages and disadvantages, as follows:

### X-PEEM

- short observation time because no sample scanning is required → acquisition time is typically several tens of seconds up to 500 seconds for one image,
- surface sensitive → probing depth is approximately a few nanometers from the sample surface,
- impossible to observe insulating materials without surface metallic coatings,
- high vacuum ~ ultrahigh vacuum condition for the sample is required for the observation,
- time-resolved measurement can be performed by combining with other excitation sources, such as the femtosecond pulsed-laser beam and the pulsed electric field.

### Scanning SX spectromicroscope

- long observation time because sample scanning is required and the low counting efficiency of characteristic X-rays in the SX region limits the measurement → typical acquisition time is a few tens of minutes up to half a day depending on the size as well as the exposure time for one pixel,
- bulk-sensitive → probing depth is about submicrometer scale,
- possible to observe insulating materials as well as a wet sample under a helium atmosphere,
- observation conditions can be controlled from a low vacuum to a helium atmosphere → in situ observations of chemical reactions will be made available by controlling the atmosphere around the sample area.

By taking advantage of the above characteristic features of the present SX spectromicroscope, we are able to explore challenging research for the future. We expect that μXRF analysis using scanning SX spectroscopy under the condition of a He atmosphere will open a new breakthrough in investigating a living attached diatom in the near future by employing a microflowing cell system that is currently under development.

## Supporting information

S1 Fig Exterior/interior 3D views of an *Arachnoidiscus* sp. frustule visualized by a laser scanning digital microscope. (A) Exterior view of a frustule taken by a laser scanning digital microscope with low spatial resolution. The image is shown in 3D view in a grayscale. The height of the frustule is approximately 25 μm. (B) Interior view of the frustule depicted in a 3D view in color.
(TIF)

S2 Fig Exterior/interior views of an *Arachnoidiscus* sp. frustule visualized by a laser scanning digital microscope. (A) Exterior view of a frustule taken by a laser scanning digital microscope with high spatial resolution. The image is shown in grayscale. (B) Interior view of a frustule visualized with high spatial resolution in grayscale. The complex structure, especially in the outer periphery, is clearly shown.
(TIF)

S3 Fig Exterior/interior view of an *Arachnoidiscus* sp. frustule taken by the SEM. (A) Exterior view of a frustule near the center region visualized in total secondary electron yield mode of SEM. (B) Same as (A) for the interior view.
(TIF)

S4 Fig X-PEEM images of an *Arachnoidiscus* sp. frustule. X-PEEM images of the diatom frustule (exterior view) obtained at four different FOVs (100 μm, 300 μm, 550 μm and 1150 μm). A UV-lamp was used as the excitation source. Larger FOV images, *i*.*e*., 550 and 1150 μm, are shown as typical examples indicating a surface charging effect. Blurred regions in the image (FOV: 1150 μm, right lower), *e*.*g*., the regions enclosed in the dotted yellow circles, show the resultant surface charging effect. In these four images, the surface charging effect is observed for larger FOVs because the acceleration voltage V_acc_ to collect the secondary electrons emitted from the sample surface is low, *e*.*g*., ~100 V (a few thousand volts) for 1150 μm (300 μm) of FOV is not sufficient to collect the secondary electrons effectively. On the other hand, the V_acc_ for the smaller FOVs is 15 kV, resulting in the effective collection of secondary electrons, which leads to imaging free of surface charging effects.
(TIF)

S5 Fig Counting efficiencies for N Kα, O Kα and elastic scattering measured as a function of oxygen concentration. The 720 eV focused SX beam extracted from the Si_3_N_4_ membrane window of the FZP chamber irradiates the resinous sample surface. The details of the sample area can be seen in Fig 1d of Ref. [28]. The N Kα and the O Kα X-rays are emitted from the air along the X-ray path as well as the sample surface and are detected by the SDD. During the measurement, the air around the sample area is gradually replaced with helium. The oxygen concentration is measured by using an oxygen monitor. At higher oxygen concentrations, most of the intensity for each line originates from the air, and the primary SX-beam hardly reaches the resinous sample due to absorption by air. As the oxygen concentration decreases, the primary SX-beam starts to reach the sample, and fluorescence X-rays from the resin are detected. At the lowest oxygen concentration, *i*.*e*., nearly a helium atmosphere, the intensity of each line from the sample approaches that measured under low-vacuum condition.
(TIF)

S6 Fig X-PEEM and CE X-PEEM images of an *Arachnoidiscus* sp. frustule recorded across the Si K-edge. The X-PEEM images recorded at the excitation energies above or at the top of the resonance, (peak) and below (pre) the Si K absorption edge. The CE X-PEEM image, *i*.*e*., div, is reproduced by peak/pre. The images are shown for two different FOVs (50 and 20 μm). At an FOV of 20 μm, the regions enclosed by the dotted yellow curves seem to be slightly affected by the surface charging effect. These results are probably due to the imperfect platinum coating prior to the X-PEEM observation.
(TIF)

S7 Fig X-PEEM and CE X-PEEM images of an *Arachnoidiscus* sp. frustule recorded across the O K-edge. Same as S6 Fig for the O K-edge.
(TIF)

S8 Fig X-PEEM and CE X-PEEM images of an *Arachnoidiscus* sp. frustule recorded across the Ca L_3_-edge. Same as S6 Fig for the Ca L_3_-edge.
(TIF)

S9 Fig μXRF single element maps of O and N in the diatom frustule. The μXRF single element maps of (a) O and (b) N visualized by measuring the O and N Kα X-rays obtained from the frustule sample of *Arachnoidiscus* sp. using a 600 eV focused SX-beam. Both μXRF maps were recorded simultaneously with a 0.5 μm pixel size and an exposure time of 1 second for one pixel, where EOI stands for energy of interest. As in the case of the μXRF single element map of Si shown in Fig 7c, the pores (*areola*) were observed at a lower counting efficiency for morphological reasons. Since we have always observed the N Kα X-ray signal from the frustule sample, we selected an EOI for N Kα X-rays for the μXRF mapping of N, although the fluorescence spectrum of N Kα X-rays is not shown in this paper. The intensity of the μXRF single element map of N was measured to be much lower than that of O, *e*.*g*., 1:75, but we could found that the map of N gave a vague outline, especially in the central region, of the morphology of the diatom frustule of *Arachnoidiscus* sp. This fact indicates that there are two possibilities for the observation of N Kα X-rays. One possibility is the inclusion of N atoms in a-SiO_2_ as an impurity, and the other possibility is that the N Kα X-rays come from the thin Si_3_N_4_ membrane window of SDD. In the latter case, the N Kα X-ray can be excited by the elastically scattered 600 eV SX-beam and the strong O Kα X-ray emission. In the near future, the latter case will be confirmed by performing a similar measurement using the SDD without the thin Si_3_N_4_ window, *i*.*e*., the windowless SDD.
(TIF)

S10 Fig Position-dependent O K-XAS spectra shown in the 3D view. The position-dependent O K-XAS spectra recorded along the horizontal black line are shown in Fig 8a. The data are essentially the same as those in Fig 8c but shown in 3D view.
(TIF)

## Abbreviations

AFM: atomic force microscopy
CE: contrast-enhanced
DOS: density of states
EDS: energy dispersive spectrometer
EELS: electron energy loss spectrometer
EOI: energy of interest
FOV: field-of-view
FWHM: full width at half maximum
FZP: Fresnel zone plate
IR: infrared
LUMO: lowest unoccupied molecular orbital
OSA: order sorting aperture
PFY: partial fluorescence yields
ROI: region of interest
SDD: silicon drift detector
SEM: scanning electron microscopy
SR: synchrotron radiation
SX: soft X-ray
TEM: transmission electron microscopy
TEY: total electron yield
TXM: transmission X-ray microscopy
UV: ultraviolet
XAS: X-ray absorption spectroscopy
XES: X-ray emission spectroscopy
XMCD: X-ray magnetic circular dichroism
XMLD: X-ray magnetic linear dichroism
X-PEEM: X-ray photoemission electron microscopy
μXRF: microprobe X-ray fluorescence
