# Supplementary figures and images for: Visualization of elemental distributions and local analysis of element-specific chemical states of an Arachnoidiscus sp. frustule using soft X-ray spectromicroscopy
Source: PLoS One. 2020 Dec 16;15(12):e0243874. doi: 10.1371/journal.pone.0243874 (PMC7743981; doi:10.1371/journal.pone.0243874)

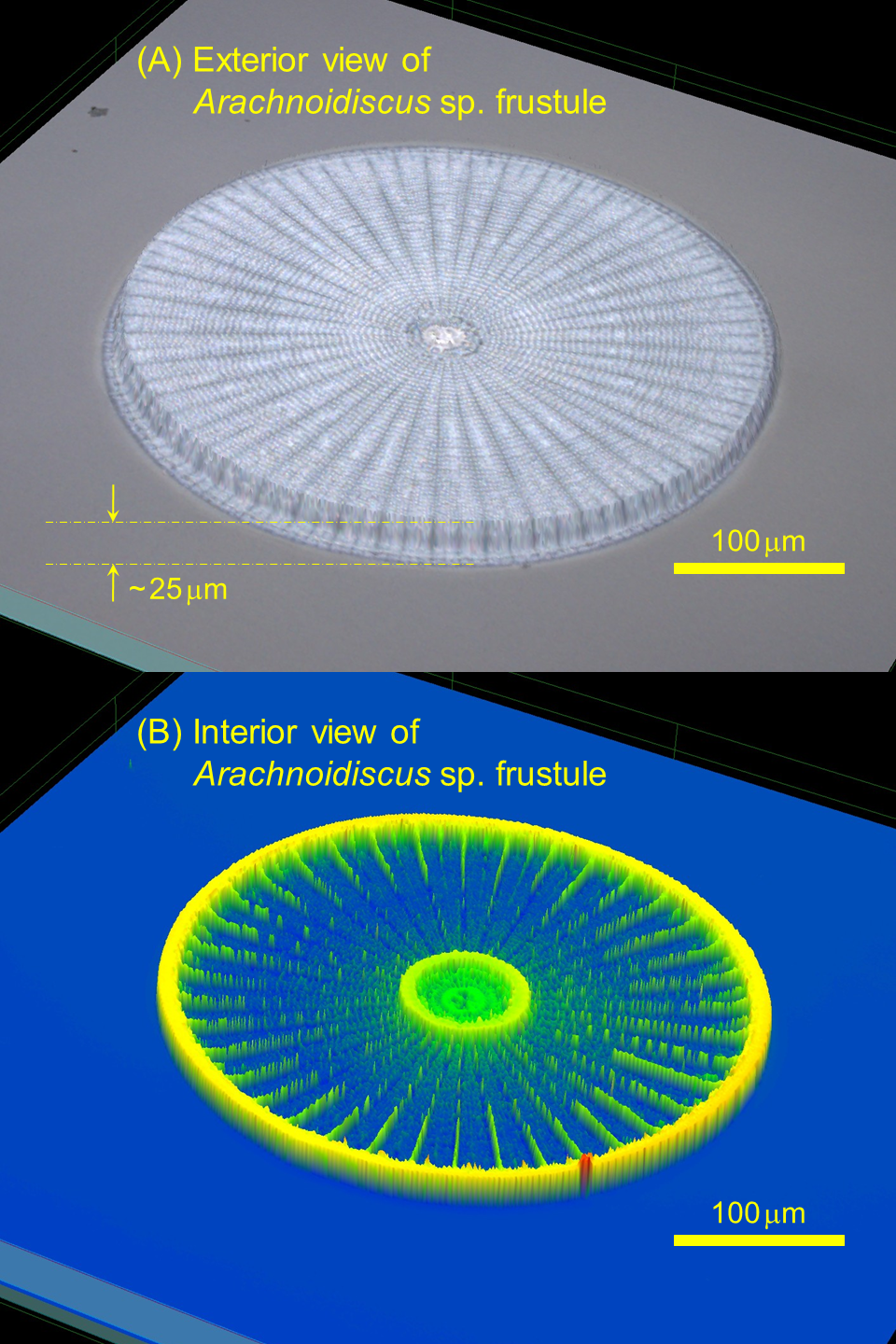

Supplement: S1 Fig — (A) Exterior view of a frustule taken by a laser scanning digital microscope with low spatial resolution. The image is shown in 3D view in a grayscale. The height of the frustule is approximately 25 μm. (B) Interior view of the frustule depicted in a 3D view in color. (TIF) [file pone.0243874.s001.tif]

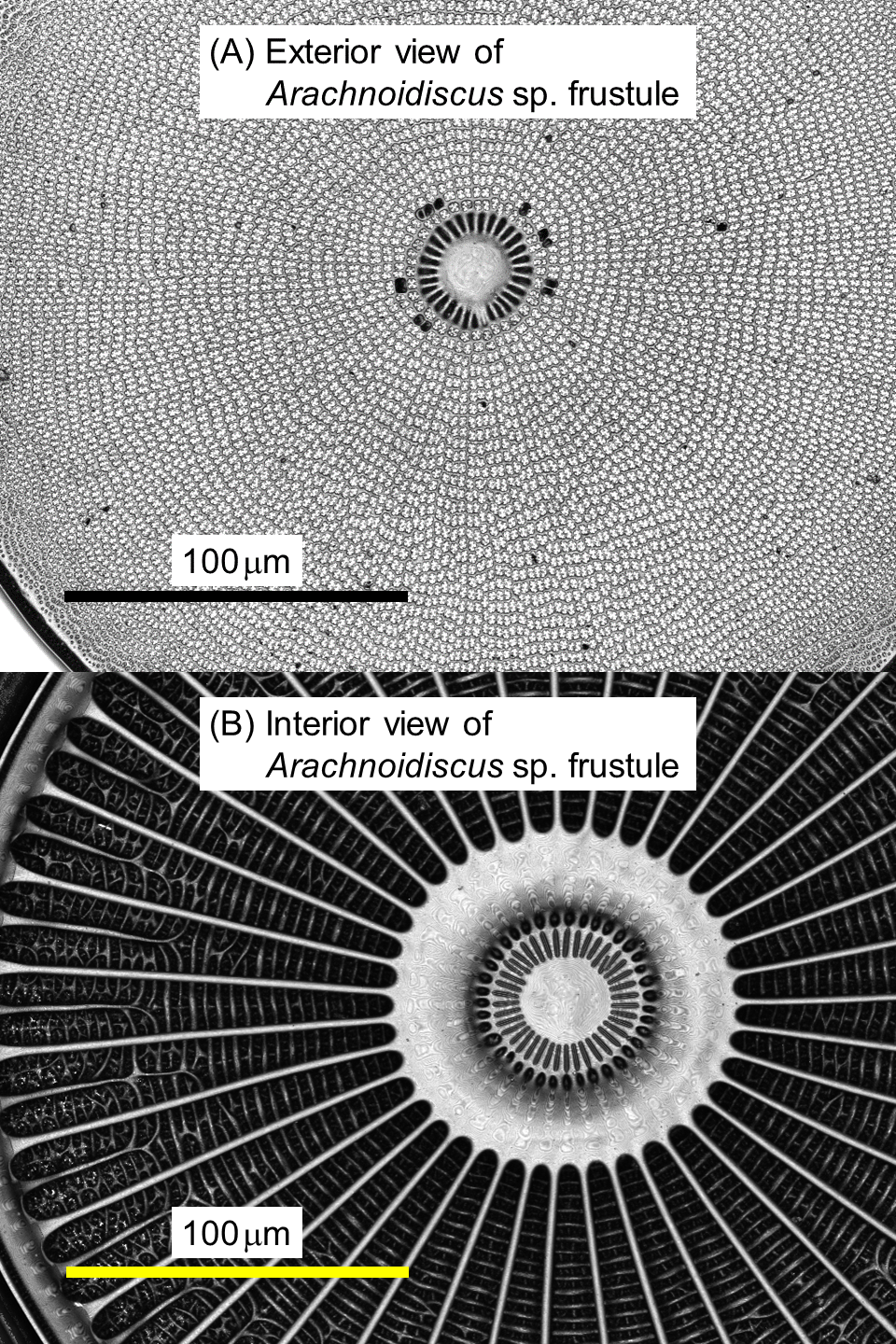

Supplement: S2 Fig — (A) Exterior view of a frustule taken by a laser scanning digital microscope with high spatial resolution. The image is shown in grayscale. (B) Interior view of a frustule visualized with high spatial resolution in grayscale. The complex structure, especially in the outer periphery, is clearly shown. (TIF) [file pone.0243874.s002.tif]

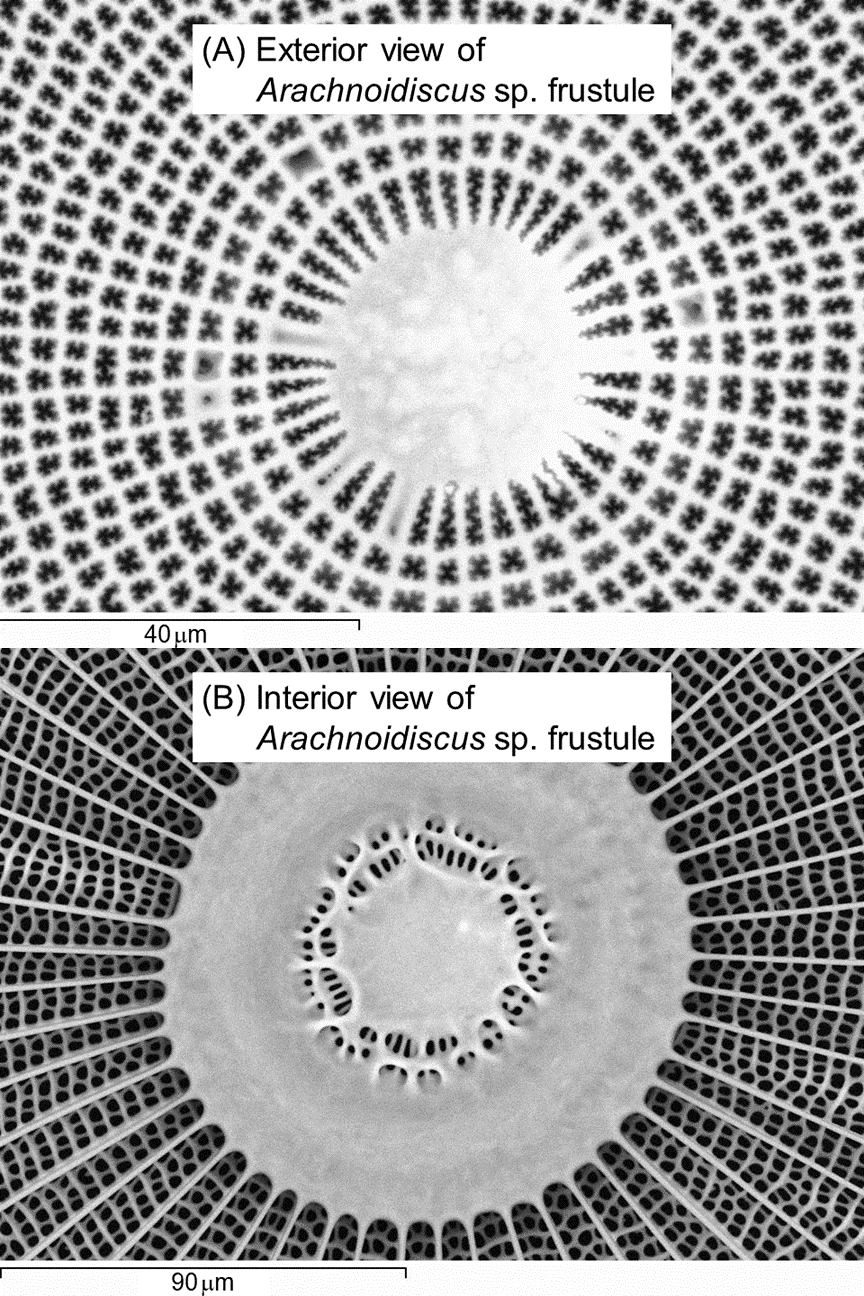

Supplement: S3 Fig — (A) Exterior view of a frustule near the center region visualized in total secondary electron yield mode of SEM. (B) Same as (A) for the interior view. (TIF) [file pone.0243874.s003.tif]

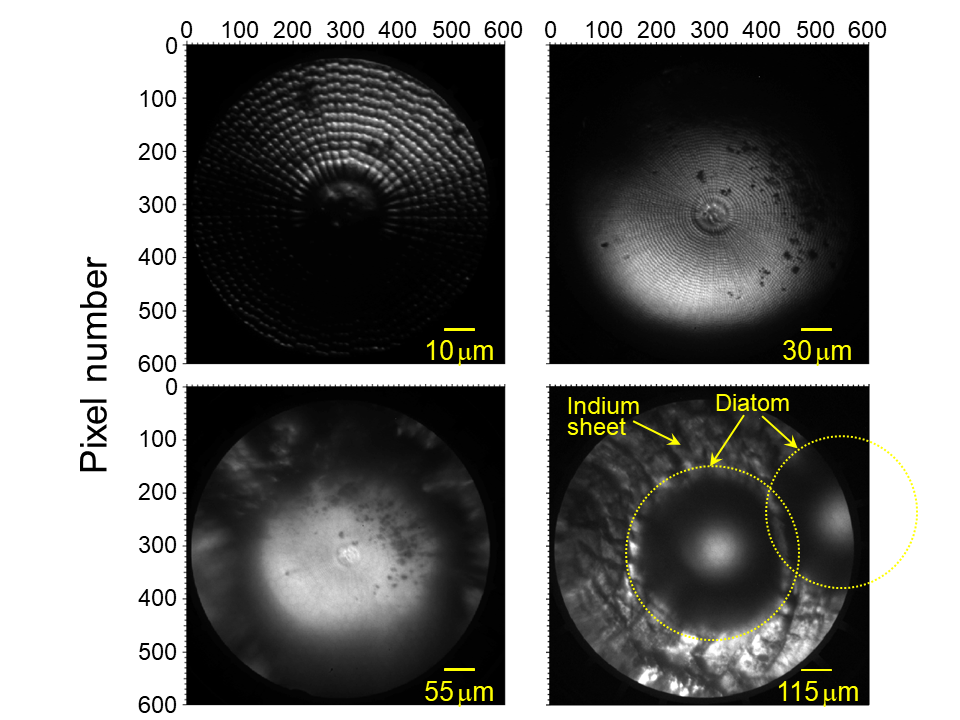

Supplement: S4 Fig — X-PEEM images of the diatom frustule (exterior view) obtained at four different FOVs (100 μm, 300 μm, 550 μm and 1150 μm). A UV-lamp was used as the excitation source. Larger FOV images, i.e., 550 and 1150 μm, are shown as typical examples indicating a surface charging effect. Blurred regions in the image (FOV: 1150 μm, right lower), e.g., the regions enclosed in the dotted yellow circles, show the resultant surface charging effect. In these four images, the surface charging effect is observed for larger FOVs because the acceleration voltage Vacc to collect the secondary electrons emitted from the sample surface is low, e.g., ~100 V (a few thousand volts) for 1150 μm (300 μm) of FOV is not sufficient to collect the secondary electrons effectively. On the other hand, the Vacc for the smaller FOVs is 15 kV, resulting in the effective collection of secondary electrons, which leads to imaging free of surface charging effects. (TIF) [file pone.0243874.s004.tif]

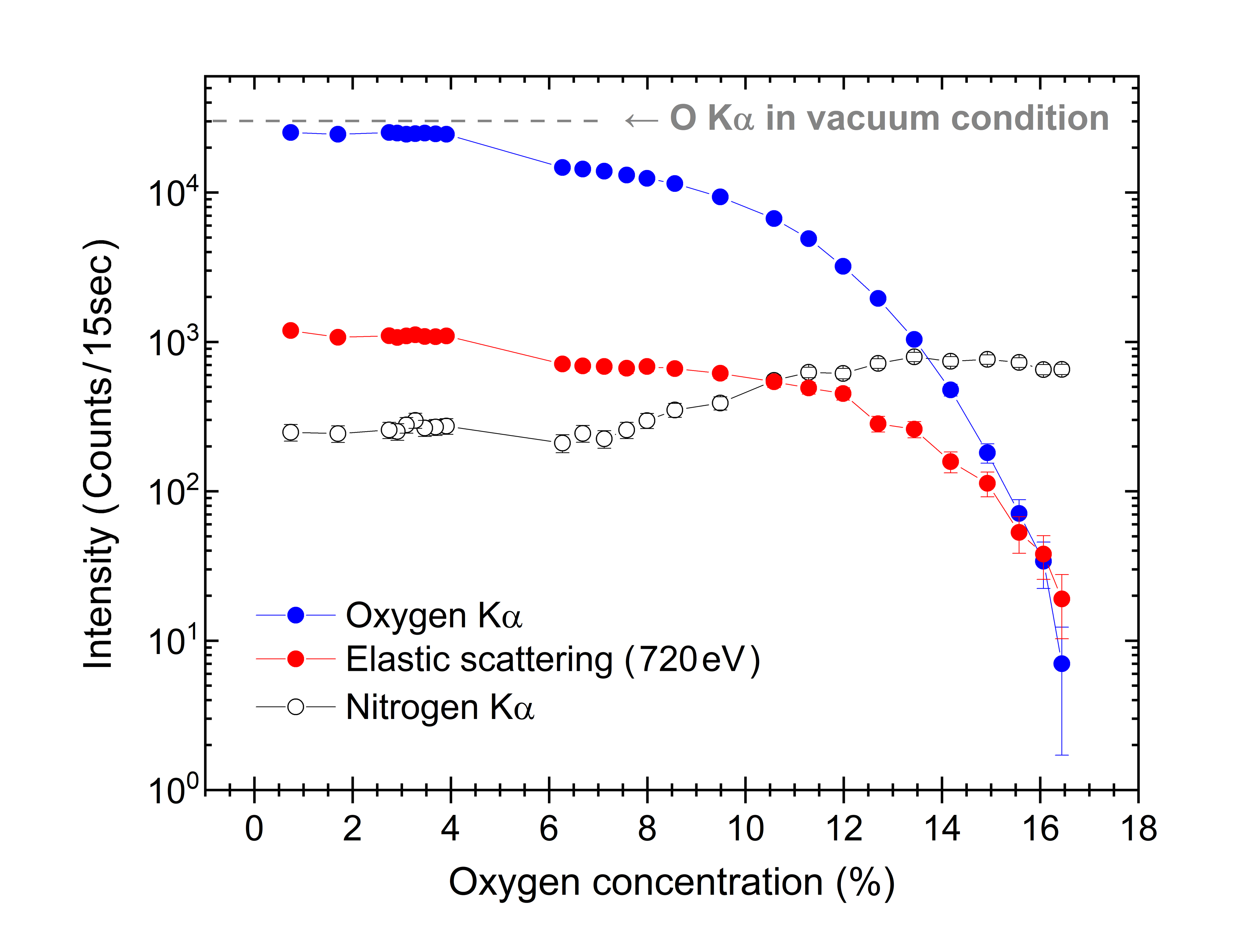

Supplement: S5 Fig — The 720 eV focused SX beam extracted from the Si3N4 membrane window of the FZP chamber irradiates the resinous sample surface. The details of the sample area can be seen in Fig 1d of Ref. [28]. The N Kα and the O Kα X-rays are emitted from the air along the X-ray path as well as the sample surface and are detected by the SDD. During the measurement, the air around the sample area is gradually replaced with helium. The oxygen concentration is measured by using an oxygen monitor. At higher oxygen concentrations, most of the intensity for each line originates from the air, and the primary SX-beam hardly reaches the resinous sample due to absorption by air. As the oxygen concentration decreases, the primary SX-beam starts to reach the sample, and fluorescence X-rays from the resin are detected. At the lowest oxygen concentration, i.e., nearly a helium atmosphere, the intensity of each line from the sample approaches that measured under low-vacuum condition. (TIF) [file pone.0243874.s005.tif]

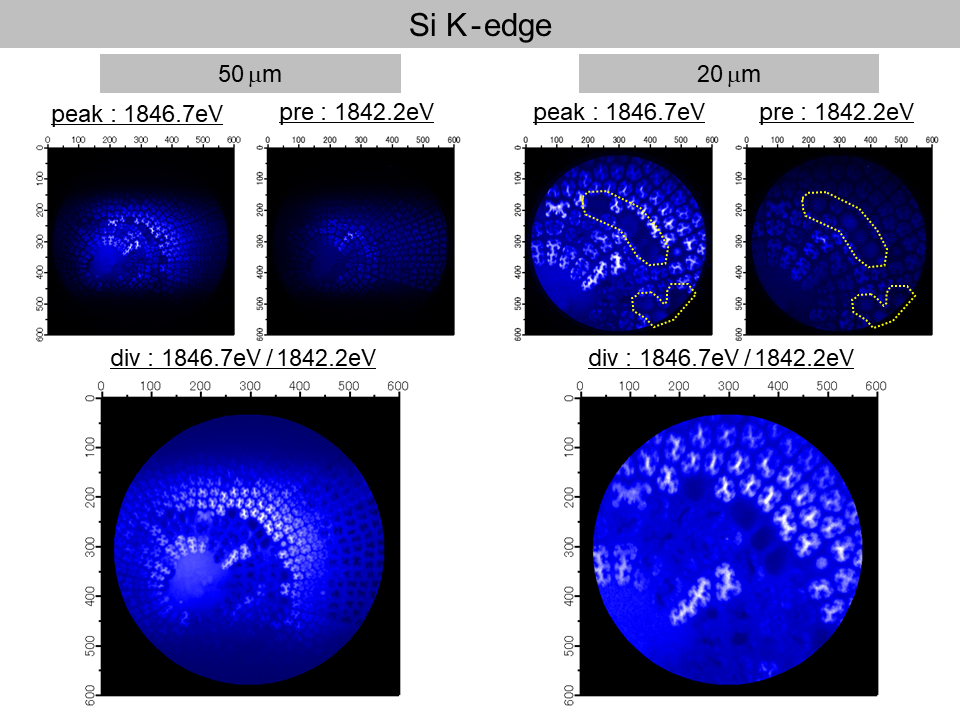

Supplement: S6 Fig — The X-PEEM images recorded at the excitation energies above or at the top of the resonance, (peak) and below (pre) the Si K absorption edge. The CE X-PEEM image, i.e., div, is reproduced by peak/pre. The images are shown for two different FOVs (50 and 20 μm). At an FOV of 20 μm, the regions enclosed by the dotted yellow curves seem to be slightly affected by the surface charging effect. These results are probably due to the imperfect platinum coating prior to the X-PEEM observation. (TIF) [file pone.0243874.s006.tif]

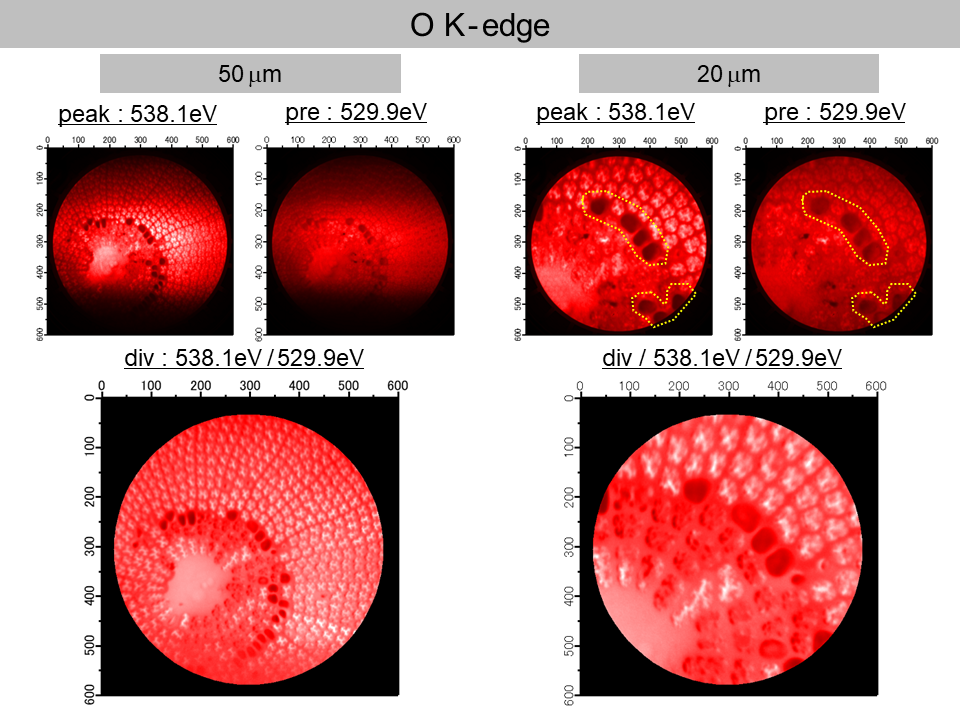

Supplement: S7 Fig — Same as S6 Fig for the O K-edge. (TIF) [file pone.0243874.s007.tif]

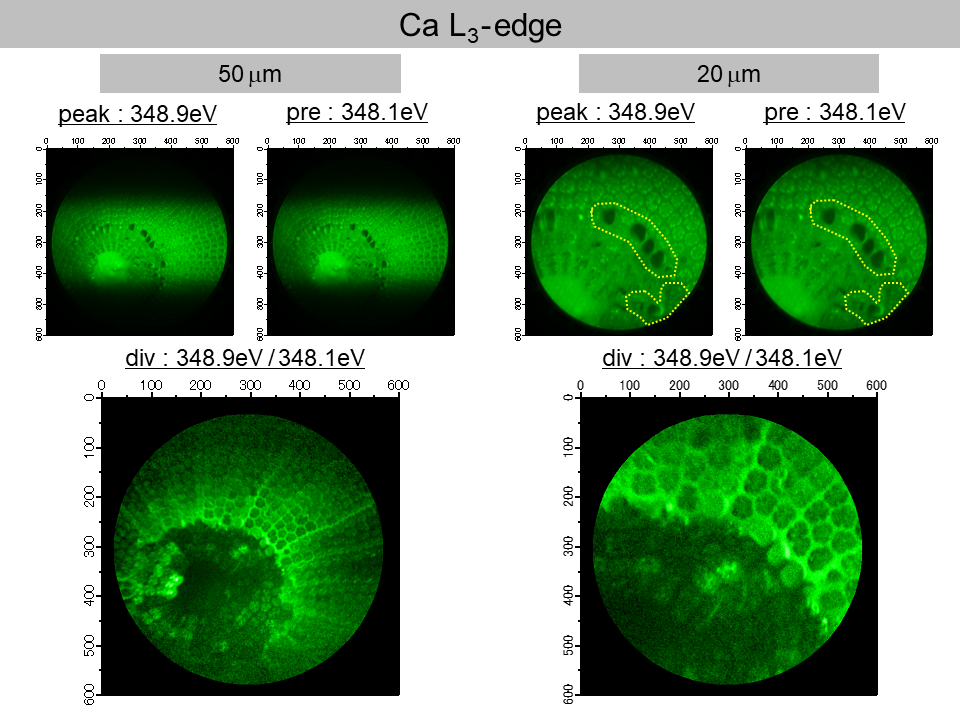

Supplement: S8 Fig — Same as S6 Fig for the Ca L3-edge. (TIF) [file pone.0243874.s008.tif]

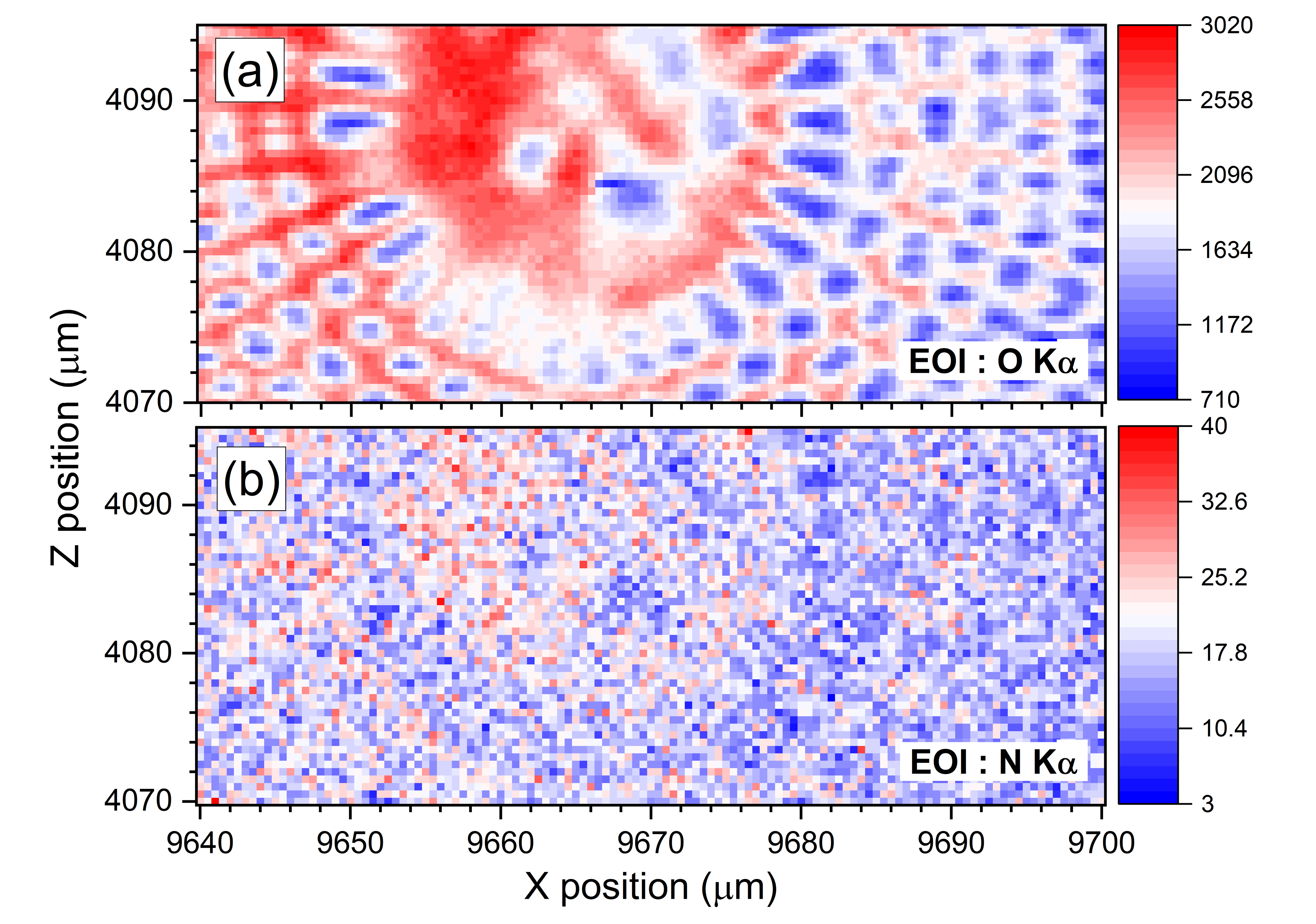

Supplement: S9 Fig — The μXRF single element maps of (a) O and (b) N visualized by measuring the O and N Kα X-rays obtained from the frustule sample of Arachnoidiscus sp. using a 600 eV focused SX-beam. Both μXRF maps were recorded simultaneously with a 0.5 μm pixel size and an exposure time of 1 second for one pixel, where EOI stands for energy of interest. As in the case of the μXRF single element map of Si shown in Fig 7c, the pores (areola) were observed at a lower counting efficiency for morphological reasons. Since we have always observed the N Kα X-ray signal from the frustule sample, we selected an EOI for N Kα X-rays for the μXRF mapping of N, although the fluorescence spectrum of N Kα X-rays is not shown in this paper. The intensity of the μXRF single element map of N was measured to be much lower than that of O, e.g., 1:75, but we could found that the map of N gave a vague outline, especially in the central region, of the morphology of the diatom frustule of Arachnoidiscus sp. This fact indicates that there are two possibilities for the observation of N Kα X-rays. One possibility is the inclusion of N atoms in a-SiO2 as an impurity, and the other possibility is that the N Kα X-rays come from the thin Si3N4 membrane window of SDD. In the latter case, the N Kα X-ray can be excited by the elastically scattered 600 eV SX-beam and the strong O Kα X-ray emission. In the near future, the latter case will be confirmed by performing a similar measurement using the SDD without the thin Si3N4 window, i.e., the windowless SDD. (TIF) [file pone.0243874.s009.tif]

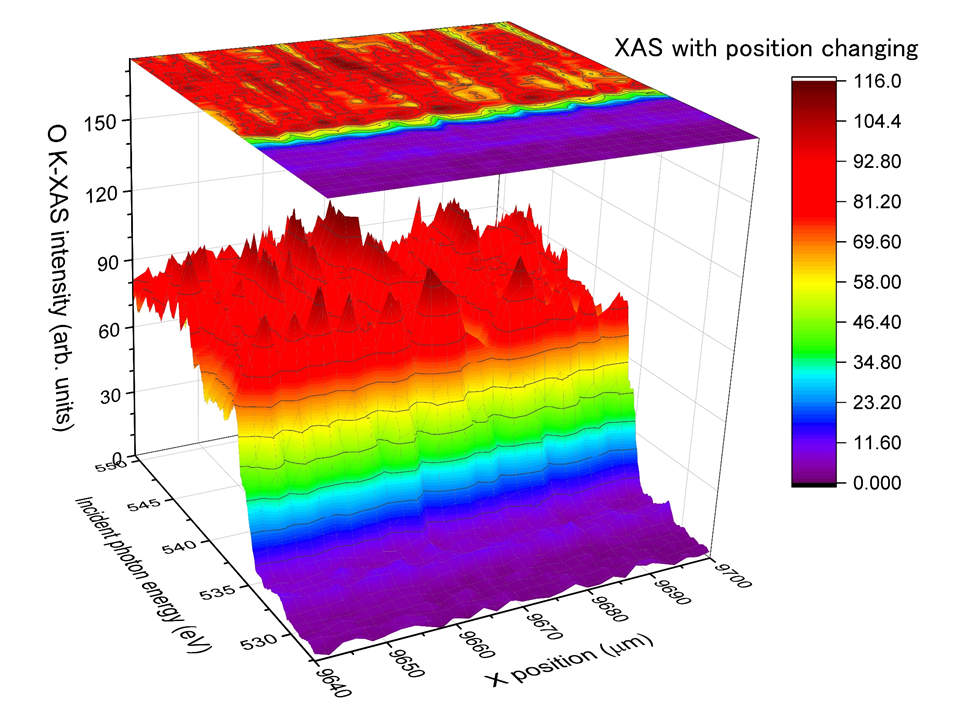

Supplement: S10 Fig — The position-dependent O K-XAS spectra recorded along the horizontal black line are shown in Fig 8a. The data are essentially the same as those in Fig 8c but shown in 3D view. (TIF) [file pone.0243874.s010.tif]
